# Fibulin-3 in plasma and pleural effusion as a biomarker of mesothelioma

**DOI:** 10.2478/raon-2025-0024

**Published:** 2025-06-16

**Authors:** Katja Adamic, Mateja Marc Malovrh, Urska Bidovec Stojkovic, Ales Rozman

**Affiliations:** 1 University Clinic of Respiratory and Allergic Diseases, Golnik, Slovenia; 2 Faculty of Medicine, University of Ljubljana, Ljubljana, Slovenia

**Keywords:** fibulin-3, asbestos, pleural effusion, mesothelioma, biomarker

## Abstract

**Background:**

Malignant pleural mesothelioma (MPM) is a global health concern linked to asbestos exposure. In Slovenia, regions with high asbestos exposure rates make MPM a significant public health issue. Although thoracoscopic biopsy is the gold standard for MPM diagnosis, its invasiveness highlights the need for reliable, non-invasive diagnostic biomarkers.

**Patients and methods:**

This prospective study evaluated the diagnostic potential of fibulin-3 as a biomarker for MPM, focusing on its ability to distinguish MPM from other pleural conditions, its association with disease stage and histological subtype, and its prognostic value for survival. Ninety patients, who underwent diagnostic thoracoscopic biopsy from January 2013 to October 2014, were included. Fibulin-3 levels in plasma and pleural effusion were measured using enzyme-linked immunosorbent assay (ELISA), and clinical data were analysed with statistical tests, including receiver operating characteristic (ROC) analysis.

**Results:**

The study cohort comprised 32 patients with MPM, 24 with metastatic pleural carcinoma, and 34 with benign pleural diseases. Plasma fibulin-3 levels were significantly elevated (p = 0.0132) in MPM patients compared to those with benign pleural effusions due to asbestos exposure, with a cut-off of 12.31 ng/mL showing 100% specificity but low sensitivity (39.39%). Elevated fibulin-3 levels in pleural effusion correlated with advanced disease (p = 0.0463) and aggressive histological subtypes (p = 0.0324). No significant survival correlation was observed.

**Conclusions:**

While plasma fibulin-3 is a highly specific biomarker for MPM, its low sensitivity limits its standalone diagnostic utility. Its potential role in risk stratification and early detection of MPM at-risk populations using combination of different and new biomarkers warrants further study.

## Introduction

Malignant pleural mesothelioma (MPM) is a rare and aggressive malignancy originating from the mesothelial cells of the pleura. Over 80% of MPM cases occur in males, predominantly those with occupational exposure to asbestos. Asbestos exposure is the primary risk factor for MPM, encountered in occupational settings, polluted environments, or through the degradation of asbestos-containing materials. The carcinogenicity of asbestos is attributed to mechanisms such as chronic pleural inflammation, generation of free radicals, interference with mitotic processes, and activation of proto-oncogenes. The latency period between asbestos exposure and the clinical onset of MPM can span several decades, contributing to late-stage diagnoses and poor prognoses.^1^

Additional risk factors for MPM include exposure to ionizing radiation, such as mantle radiation therapy for Hodgkin’s lymphoma, and germline mutations in the BRCA1-associated protein 1 (BAP1) gene.^2^ Additionally, evidence suggests that genetic variability in antioxidative defense and DNA repair, along with gene-gene interactions, may contribute to the development of malignant mesothelioma.^3^

Clinically, MPM often manifests with pleural effusion or dull, non-specific chest pain, which complicates its differentiation from asbestos-related pleuritis or other pleural pathologies. Positron emission tomography (PET) scans with 18F-fluorodeoxyglucose (18F-FDG) demonstrate high sensitivity (88–95%) in detecting MPM and also hold prognostic value.^4^

Histologically, MPM is classified into three primary subtypes: epithelioid (the most common), sarcomatoid (the most aggressive), and biphasic. Despite advancements in therapeutic approaches, MPM remains largely incurable, with a poor overall prognosis. However, recent data indicate that the introduction of contemporary systemic treatments has led to a modest improvement in overall median survival by a few months.^5^ Additionally, a slight increase in 1-year survival from 38% to 40% and in 3-year survival from 7% to 10% has been observed.^6^ Moreover, the proportion of patients over 65 years of age has risen from 41.8% to 54.6%, accompanied by a slight increase in the median age at diagnosis from 75 to 76 years^6^.

Managing MPM requires a multidisciplinary approach that integrates considerations of histological subtype, disease stage, patient age, comorbidities, performance status, and individual preferences.^7^ Multimodal therapies - encompassing chemotherapy, radiotherapy, and immunotherapy - are the cornerstone of treatment, while mutilating radical surgeries are increasingly being abandoned.

Immunotherapy has emerged as a promising treatment modality for MPM. The combination of nivolumab and ipilimumab, as demonstrated in the phase III CheckMate trial, is now FDAapproved as the first-line treatment for advanced-stage MPM and represents a significant therapeutic milestone.^8,9^

The identification of biomarkers for MPM has garnered substantial research interest, aiming to achieve three primary objectives: (1) screening at-risk populations, including asbestos-exposed individuals and genetically predisposed relatives; (2) enhancing diagnostic accuracy in patients presenting with pleural abnormalities such as effusions or pleural thickening; and (3) monitoring therapeutic response and prognosis. Soluble or pleural fluid biomarkers offer the potential to reduce the need for invasive diagnostic procedures, particularly in patients with poor performance status. The 2018 British Thoracic Society guidelines advocate biomarker testing for patients with suspicious cytology who are unfit for invasive diagnostics but caution against their use in isolation for screening, diagnosis, or prognostic purposes.^10^ Similarly, the 2020 European Respiratory Society (ERS) guidelines do not endorse routine biomarker testing, such as mesothelin, for these purposes without robust supporting evidence.^11^

Effective screening methods should prioritize minimally invasive, cost-effective techniques with high specificity to minimize false positives and associated patient anxiety.^12^

### Fibulin-3 as a biomarker in pleural fluid

Fibulin-3 is a glycoprotein encoded by the epidermal growth factor-containing fibulin-like extracellular matrix protein 1 gene, implicated in cellular proliferation and migration.^13,14^ Typically expressed at low levels, fibulin-3 is overexpressed in various malignancies, including MPM, and is secreted into body fluids. Elevated fibulin-3 concentrations in pleural effusions have been proposed as a distinguishing marker for MPM relative to benign pleural conditions. Initial studies reported high diagnostic accuracy, with an area under the receiver operating characteristic curve (AUC_ROC_) of 0.93 and optimal sensitivity and specificity thresholds between 346 ng/mL and 378 ng/mL.^15^ However, subsequent investigations have yielded conflicting results, with some reporting comparable fibulin-3 levels in pleural effusions from MPM and other pleural diseases.^16^ A meta-analysis by Schillebeeckx *et al*. estimated an overall AUC_ROC_ of 0.68 (95% CI: 0.50–0.87).^17^

Comparative analyses suggest that mesothelin outperforms fibulin-3 as a diagnostic marker in pleural effusions and plasma, while fibulin-3 may hold greater prognostic value.^18,19^ Evidence indicates that fibulin-3 promotes malignant behaviour in mesothelial cells, with knockdown studies showing reductions in cell viability, clonogenicity, invasiveness, and chemoresistance.^20^

### Fibulin-3 as a blood biomarker

The diagnostic potential of plasma fibulin-3 was first highlighted in a 2012 study by Pass *et al*., which reported an AUC_ROC_ of 0.99, sensitivity of 95%, and specificity of 95% in distinguishing MPM patients from healthy asbestos-exposed controls and patients with other malignancies.^15^ Subsequent studies, however, have reported lower diagnostic accuracy and inconsistent findings.^19,21–23^

A recent meta-analysis demonstrated an overall AUC_ROC_ of 0.91 for plasma fibulin-3, although head-to-head comparisons with mesothelin and soluble mesothelin-related peptides (SMRP) yielded inconclusive results.^17,19,24^ Current evidence suggests that serum/plasma fibulin-3 is not a reliable prognostic marker or predictor of treatment response in MPM.^25,26^ Nevertheless, fibulin-3 remains a promising molecular target for therapeutic intervention, with anti-fibulin-3 strategies under active investigation.^27^

### Study objectives

In this study, we sought to evaluate the diagnostic utility of fibulin-3 in pleural mesothelioma. To date, no research in Slovenia has specifically explored fibulin-3 as a diagnostic marker for MPM. A previous study in 2015 assessed its prognostic and therapeutic response potential.^28^

Our objectives were to quantify fibulin-3 concentrations in plasma and pleural effusions across various diseases included in the differential diagnosis of MPM, assess its discriminatory value, and examine associations between fibulin-3 levels (systemic and pleural) with disease stage and patient survival.

## Patients and methods

### Cohort and subject definition

This prospective clinical study included 90 patients undergoing diagnostic thoracoscopy for the evaluation of pleural disease at the University Clinic for Pulmonary Diseases and Allergy Golnik, Slovenia, between January 1, 2013, and October 15, 2014. Based on final diagnoses, patients were categorized into four distinct groups:

– *Group A:* Patients with benign asbestos-related pleural disease.
– *Group M:* Patients diagnosed with malignant pleural mesothelioma (MPM).
– *Group C:* Patients with metastatic pleural carcinomatosis.
– *Group F:* Patients with benign pleural effusions of non-asbestos-related causes, including fibroproductive, post-traumatic, lymphocytic, eosinophilic pleuritis, pleuritis post-cardiac surgery, and parapneumonic effusions.

All participants provided written informed consent after being fully briefed on the study objectives and procedures. Inclusion criteria were the presence of exudative pleural effusion requiring thoracoscopy for diagnosis, age over 18 years, and willingness to participate. The sole exclusion criterion was refusal to participate.

### Fibulin-3 concentration measurement

Concentrations of fibulin-3 in plasma and pleural effusion samples were measured using a commercially available enzyme-linked immunosorbent assay (ELISA) kit (Cloud-Clone Corp., Houston, TX, USA). All samples were processed and analysed according to the manufacturer’s instructions.

### Statistical analysis

Statistical analyses were performed using GraphPad Prism version 10.0, Microsoft Excel 2016, and IBM SPSS Statistics version 27 software. Descriptive statistics were applied to summarize the data. Continuous variables were reported as mean ± standard error (SE) for normally distributed data and as median with interquartile range (IQR) for non-normally distributed data. Nonparametric t-tests and Mann-Whitney U tests were utilized for the analysis of independent samples. Receiver operating characteristic (ROC) curves were employed to evaluate the diagnostic accuracy of fibulin-3, with the area under the curve (AUC) calculated to quantify diagnostic performance. A 95% confidence interval (CI) was used to determine if the AUC was significantly greater than 0.5. A p-value < 0.05 was considered statistically significant.

### Ethical consideration

The study complied with the principles of the Helsinki-Tokyo Declaration and received ethical approval from the National Medical Ethics Committee of the Republic of Slovenia (KME No. 206/03/13).

## Results

### Patient characteristics

Over a 22-month period, from January 2013 to October 2014, a total of 90 patients were enrolled in the study, with 70 of them being male (77.8%). The ages of the participants at the time of sample collection ranged from 36 to 80 years, with an average age of 64.44 years (SE = 1.14 years).

**Table 1.** Characteristics of patients

	A (n = 13)	M (n = 32)	C (n = 24)	F (n = 21)
**Age (mean, SE)**	68.1 (2.4)	63.7 (1.9)	63.1 (1.9)	63.8 (3.0)
**Male gender – n (%)**	12 (92.3)	24 (75.0)	14 (58.3)	20 (95.2)
**Smoking status – n (%)**				
Active smoker	1 (7.6)	7 (21.9)	2 (8.3)	3 (14.3)
Former smoker	5 (38.5)	6 (18.8)	11 (45.8)	11 (52.4)
Non-smoker	7 (53.8)	17 (53.1)	8 (33.3)	4 (19.0)
**Asbestos exposure – n (%)**	13 (100.0)	19 (59.4)	2 (8.3)	4 (19.0)

1 A = benign pleural disease related to asbestos; C = metastatic carcinomatosis; F = benign pleural effusion of other aetiologies; M = malignant mesothelioma; n = number of patients; SE = standard error

The underlying causes of pleural effusion or pleural changes were classified as follows:

– *MPM (Group M):* 32 patients (35.6%)
– *Metastatic pleural carcinomatosis (Group C):* 24 patients (26.7%)
– *Benign pleural effusion:* 34 patients (37.8%)
– Among those with benign pleural effusion:
– *Asbestos-related pleural disease (Group A):* 13 patients (14.4%)
– *Benign pleural effusions of other aetiologies (Group F):* 21 patients (23.3%)
  ◦ 15 with nonspecific fibroproductive or chronic pleuritis
  ◦ 3 with residual parapneumonic effusion
  ◦ 1 with eosinophilic pleuritis of unknown origin
  ◦ 1 with pleuritis due to injury
  ◦ 1 with pleuritis following cardiac surgery.

### Diagnostic value of fibulin-3 concentration in plasma and pleural effusion

**Figure 1.**
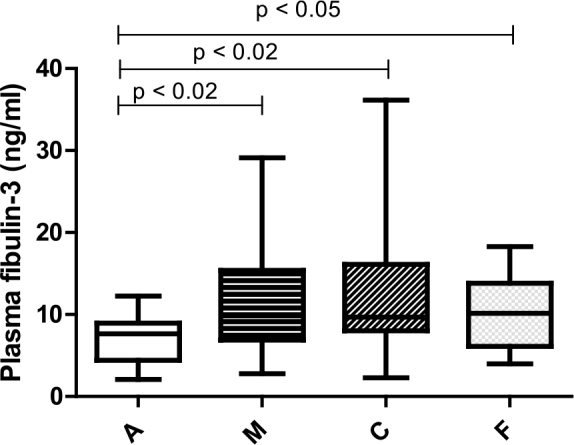
Plasma fibulin-3 levels by group. A = benign pleural disease related to asbestos; C = metastatic carcinomatosis; F = benign pleural effusion of other aetiologies; M = mesothelioma

Given the non-normal distribution of fibulin-3 concentrations in both plasma and pleural effusion samples, the Mann-Whitney test was employed for analysis, with results expressed as median and interquartile range (IQR). Fibulin-3 concentrations in pleural effusion could not be measured for 10 patients (4 from Group A and 6 from Group M) due to the absence of effusion during thoracoscopy. Additionally, in 4 cases, pleural effusion samples were not delivered to the laboratory promptly for unspecified reasons. The fibulin-3 values for the different groups are presented in Table 2 and Figure 1.

**Table 2.** Fibulin-3 values, presented with the median and interquartile range (IQR)

	A (n = 13)	M (n = 32)	C (n = 24)	F (n = 21)
**Fibulin-3 value (ng/mL)**				
**Plasma**	7.66 (4.43–8.96)	9.91 (6.08–15.83)	9.21 (7.97–15.93)	10.15 (6.125–13.83)
**Pleural effusion**	83.67 (15.71–87.21)	78.61 (44.4–94.37)	81.25 (35.86–95.45)	79.64 (41.43–86.99)

We found a statistically significant difference in plasma fibulin-3 levels between patients with asbestos-related pleural changes (Group A) and those with mesothelioma (Group M), with higher levels observed in the mesothelioma group (p = 0.0132). Additionally, fibulin-3 levels in Group A were significantly lower compared to patients with metastatic pleural carcinomatosis (p = 0.0251)and those with benign pleural effusion of other aetiologies (p = 0.0452). However, no significant differences in plasma fibulin-3 levels were observed between Group M and Groups C and F.

**Figure 2.**
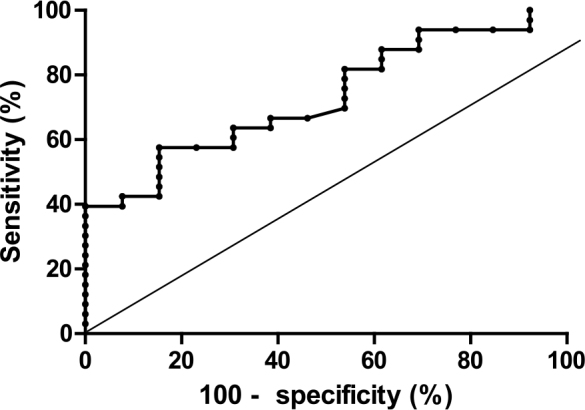
Receiver-operating characteristic curve (ROC) for plasma fibulin-3 in distinguishing Group M (mesothelioma) and Group A (benign pleural disease related to asbestos).

The area under the curve (AUC) for plasma fibulin-3 in distinguishing mesothelioma (Group M) from asbestos-related benign pleural disease (Group A) was 0.78 (p < 0.02) (Figure 2). A plasma fibulin-3 level greater than 12.31 ng/ml demonstrated a specificity of 100% and a sensitivity of 39.39% for detecting mesothelioma.

No differences in fibulin-3 levels in pleural effusion were detected between the selected groups. Additionally, there was no statistically significant correlation between fibulin-3 levels in plasma and those in pleural effusion.

### Fibulin-3 Levels by Mesothelioma Type, Stage, and Survival

Among the 32 patients with MPM, 23 were diagnosed with epithelioid mesothelioma, 2 with sarcomatoid mesothelioma, and 7 with biphasic (mixed) mesothelioma (Table 3).

**Table 3.** Fibulin-3 levels by mesothelioma type, shown with median and interquartile range

	Epitheloid type	Biphasic and sarcomatoid type
**Fibulin-3 value (ng/mL)**		
**Plasma**	9.96 (7.66-16.65)	8.62 (5.91–13.26)
**Pleural effusion**	66.15 (42.72-93.64)	92.3 (82.6–105.2)

Fibulin-3 levels in pleural effusion were significantly higher in patients with biphasic or sarcomatoid mesothelioma compared to those with epithelioid mesothelioma, as determined by the Mann-Whitney test (p = 0.0324). No significant differences in plasma fibulin-3 levels were observed between these groups.

**Figure 3.**
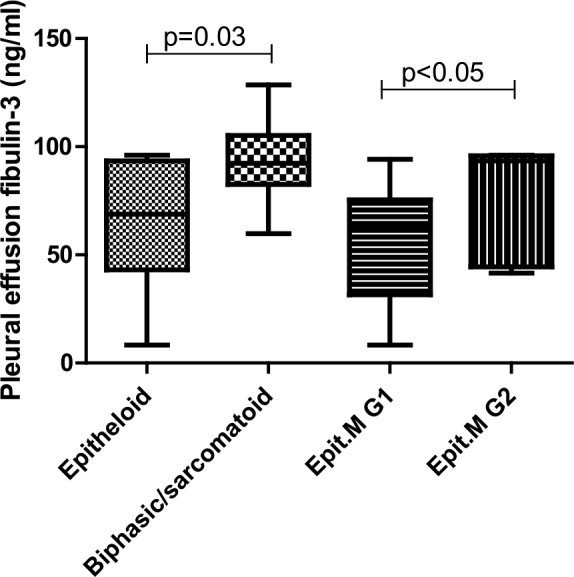
Fibulin-3 levels according to stage and type of malignant pleural mesothelioma (MPM). Epit. M = epithelioid mesothelioma; G1 = Group G1, lower stage of MPM; G2 = Group G2, higher stage of MPM

Among patients with epithelioid mesothelioma, those were further categorized into two stages: Group G1 (TNM stage IA to T2N0M0, n = 11) and Group G2 (more advanced stages, n = 7). Patients in the higher stage Group G2 had significantly higher fibulin-3 levels in pleural effusion, with a p-value of 0.0463 (Mann-Whitney test) (Figure 3).

**Figure 4.**
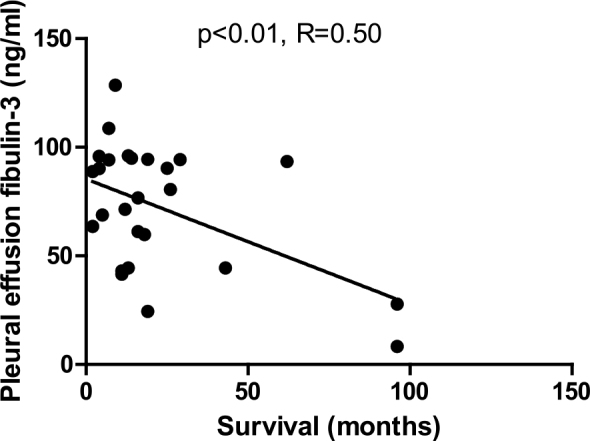
Correlation between fibulin-3 levels in pleural effusion and patient survival in mesothelioma patients.

The median survival time for patients with mesothelioma was 14.1 months, with an interquartile range of 7.7 to 26.1 months. We found significant correlation between fibulin-3 levels in pleural effusion and patient survival in mesothelioma patient subgroup, whereas there was no correlation between plasma fibulin-3 levels and mesothelioma patients’ survival (Figure 4).

## Discussion

### Fibulin-3 in plasma

This study evaluated fibulin-3 levels in plasma among patients with malignant pleural mesothelioma (MPM), benign pleural conditions, and metastatic pleural carcinomatosis, addressing the diagnostic challenge posed by overlapping clinical presentations and examination results.

A significant difference in plasma fibulin-3 levels was observed between MPM patients (median = 9.91 ng/mL) and those with benign asbestos-related pleural conditions (median = 7.66 ng/mL). Despite the study’s limited sample size, the calculated AUC_ROC_ of 0.78 suggests moderate diagnostic ability of fibulin-3 for distinguishing MPM from benign asbestos-related pleural conditions. The optimal cut-off value of 12.31 ng/mL yielded 100% specificity and 39.39% sensitivity.

These findings align with prior studies reporting plasma fibulin-3 as a diagnostic marker for MPM, with reported AUC_ROC_ values ranging from 0.81 to 0.99, demonstrating high specificity and sensitivity.^21,29,30^ For example, Jiang *et al*. found elevated plasma fibulin-3 levels in MPM patients compared to asbestos-exposed individuals, with cut-off values ranging from 15.3 to 15.9 ng/mL and sensitivities of 86.7% to 93.6%.^29^ However, differences in methodologies, patient populations, and cut-off thresholds across studies complicate direct comparisons.^15^

The variability in plasma fibulin-3 levels may reflect differences in patient characteristics or disease biology. While some studies have reported higher plasma fibulin-3 levels in early-stage MPM compared to healthy controls^13,15,29^, it remains unclear whether these levels are a cause or consequence of mesothelioma progression, and if fibulin-3 plays a role in disease development. Elevated plasma fibulin-3 levels have also been reported in pulmonary asbestosis, consistent with findings of increased fibulin-3 expression in lung tissues and fibroblasts exposed to asbestos.^29,30^ However, the absence of correlations between the duration of asbestos exposure and plasma fibulin-3 levels in two prior studies is notable.^13,15^ Our study could neither address this relationship due to insufficient exposure data and the lack of healthy controls.

Interestingly, plasma fibulin-3 levels in asbestos-related pleural conditions were lower than those in metastatic pleural carcinomatosis (median = 9.21 ng/mL). Elevated fibulin-3 levels have been associated with malignancies such as pancreatic cancer and glioblastoma, suggesting a role in tumour progression. Conversely, lower fibulin-3 levels have been reported in other cancers, suggesting that high fibulin-3 levels in our study might reflect advanced malignant disease.^31–34^

The unexpectedly higher plasma fibulin-3 levels in benign pleural effusions of other causes (median = 10.15 ng/mL) compared to asbestos-related conditions require further investigation, particularly given the heterogeneity of this group, which included cases of chronic pleural inflammation. The lack of significant differences in plasma fibulin-3 levels between MPM and other groups is in contrast with findings from Pass *et al*.^15^

### Fibulin-3 in pleural effusion

Fibulin-3 levels in pleural effusion were expected to better reflect the biological behaviour of MPM, according to Pass *et al*.^15^ However, our analysis did not demonstrate significant differences between MPM patients and those with benign pleural effusions from asbestos exposure or other causes. This aligns with previous studies reporting limited diagnostic utility of pleural effusion fibulin-3 levels for MPM.^16,19,21^ Elevated fibulin-3 expression in MPM tumour cells likely contributes to increased plasma and pleural effusion levels, yet no significant correlation was found between fibulin-3 levels in these two compartments in our study, consistent with earlier findings.^15,21^

Soluble mesothelin-related peptide (SMRP) remains a more reliable biomarker for distinguishing benign from MPM pleural effusions, as supported by Kovac *et al*.^28^. In contrast, Pass *et al*. reported higher pleural effusion fibulin-3 levels in MPM patients, a finding not corroborated by our data.^15^

### Fibulin-3 and MPM subtypes and stages

Among the 32 MPM patients in our cohort, 23 had epithelioid mesothelioma, 2 had sarcomatoid, and 7 had biphasic types, consistent with the established distribution of histological subtypes.^35^ Significantly higher pleural effusion fibulin-3 levels were observed in sarcomatoid and biphasic subtypes compared to epithelioid mesothelioma. Creaney *et al*., have linked elevated pleural effusion fibulin-3 levels with poor survival outcomes, although we did not observe this association in our study.^19^ Similar results showing that plasma fibulin-3 levels are not influenced by histological type have been noted in both Slovenian and international studies.^15,28^

Fibulin-3 levels in pleural effusion were significantly higher in patients with advanced-stage MPM, suggesting a correlation with tumour burden. Pass *et al*. found significant differences in fibulin-3 levels between stage I or II and stage III or IV patients.^15^ However, our study did not find significant differences in plasma fibulin-3 levels by mesothelioma stage, suggesting that plasma fibulin-3 may be more indicative of early mesothelial cell transformation. We did not find a correlation between plasma and pleural effusion fibulin-3 levels, consistent with previous studies.^15^

### Fibulin-3 and survival

Research into the prognostic value of fibulin-3 in MPM remains limited.^28^ While some Slovenian and international studies suggest that plasma fibulin-3 may have value in assessing disease progression and survival, our results did not demonstrate a significant correlation between plasma fibulin-3 levels and survival outcomes.^19,21,28,36^ In contrast, higher pleural effusion fibulin-3 levels were associated with worse survival in our cohort, consistent with prior findings.

### Limitations

This study has several limitations, including the absence of healthy controls or individuals with documented asbestos exposure without pleural disease. The small sample size, particularly for fibulin-3 subgroup analyses, and the lack of comprehensive asbestos exposure data further restrict the generalizability of our findings. Despite these limitations, our results contribute to understanding fibulin-3’s potential as a biomarker for MPM.

## Conclusions

Our findings indicate that plasma fibulin-3 levels can help distinguish MPM from benign asbestos-related pleural conditions, even in early disease stages. However, its low sensitivity and the absence of significant differences in plasma fibulin-3 levels between MPM and other groups (metastatic carcinomatosis of pleura, benign non-asbestos related pleuritis) limit its diagnostic value as a standalone marker. Pleural effusion fibulin-3 levels did not significantly differ between MPM and benign conditions but were elevated in aggressive MPM subtypes and advanced disease stages.

Our study did not support the hypothesis of a negative correlation between fibulin-3 levels in pleural effusions and survival. Fibulin-3 levels were not associated with survival, stage, or type of MPM. Despite these insights, fibulin-3 holds potential as an early diagnostic marker for MPM but further research is needed to refine its diagnostic and prognostic roles relative to established markers such as SMRP. Future studies with larger, well-characterized cohorts are essential to validate these findings and explore the clinical utility of fibulin-3 in early diagnosis, and therapeutic monitoring of at-risk and affected populations.
